# Navigating the learning curve: assessing caseload and comparing outcomes before and after the learning curve of computer-navigated total hip arthroplasty

**DOI:** 10.1007/s11701-024-01855-4

**Published:** 2024-03-02

**Authors:** Christian J. Hecht II, Joshua R. Porto, Parshva A. Sanghvi, Yasuhiro Homma, Peter K. Sculco, Atul F. Kamath

**Affiliations:** 1 Department of Orthopaedic Surgery, Center for Hip Preservation, Orthopaedic and Rheumatologic Institute, Cleveland Clinic Foundation, 9500 Euclid Avenue, Mail Code A41, Cleveland, OH 44195 USA; 2 Department of Medicine for Orthopaedics and Motor Organs, Juntendo University Graduate School of Medicine, Bunkyo-ku, Tokyo, Japan; 3 Department of Orthopaedics, Faculty of Medicine, Juntendo University, Bunkyo-ku, Tokyo, Japan; 4 Department of Orthopaedic Surgery, Hospital for Special Surgery, New York, NY 10021 USA

**Keywords:** Computer navigation, Total hip arthroplasty, Learning curve, CUSUM analysis

## Abstract

**Purpose:**

Computer-navigated (CN) total hip arthroplasty (THA) offers improved acetabular component placement and radiographic outcomes, but inconsistent assessment methods of its learning curves render the evaluation of adopting a novel platform challenging. Therefore, we conducted a systematic review to assess the learning curve associated with CN-THA, both tracking a surgeon's performance across initial cases and comparing their performance to manual THA (M-THA).

**Methods:**

A search was conducted using PubMed, MEDLINE, EBSCOhost, and Google Scholar on June 16, 2023 to find research articles published after January 1, 2000 (PROSPERO registration: CRD4202339403) that investigated the learning curve associated with CN-THA. 655 distinct articles were retrieved and subsequently screened for eligibility. In the final analysis, nine publications totaling 847 THAs were evaluated. The Methodological Index for Nonrandomized Studies (MINORS) tool was utilized to evaluate the potential for bias, with the mean MINORS score of 21.3 ± 1.2.

**Results:**

CN-THA showed early advantages to M-THA for component placement accuracy and radiographic outcomes but longer operative times (+ 3− 20 min). There was a learning curve required to achieve peak proficiency in these metrics, though mixed methodologies made the required caseload unclear.

**Conclusions:**

CN-THA offers immediate advantages to M-THA for component placement accuracy and radiographic outcomes, though CN-THA’s advantages become more pronounced with experience. Surgeons should anticipate longer operative times during the learning curve for CN-THA, which lessen following a modest caseload. A more thorough evaluation of novel computer-navigated technologies would be enhanced by adopting a more uniform method of defining learning curves for outcomes of interest.

*Registration* PROSPERO registration of the study protocol: CRD42023394031, 27 June 2023.

## Introduction

Accurate placement of the acetabular component is crucial for favorable outcomes after total hip arthroplasty (THA), as malposition of the implant is a prominent cause of complications and revision [1–4]. Traditionally, surgeons rely on intraoperative landmarks to guide placement, a challenging feat that has become increasingly difficult with the popularity of minimally invasive procedures [5]. Therefore, manual techniques for attaining consistent and accurate component placement and restoration of leg length and offset are challenging. This has created a growing demand for surgical technologies, such as computer-navigated (CN) THA platforms, which have demonstrated improved placement of the acetabular component and radiographic outcomes compared to manual THA (M-THA) [6–9]. However, much like the acquisition of any other surgical skill, surgeons face a learning curve upon adoption of surgical technologies [10–13].

Surgical learning curves have received growing interest in recent years, as studies continue to indicate substantial implications related to cost-effectiveness, clinical outcomes, and patient safety [14–19]. The surgical learning curve was initially described by Luft et al. [20] as having four stages: (1) at the onset of training, a sharp uprise in the measured outcome; (2) period of diminishing returns with slight improvements in the outcome; (3) plateau exhibiting no further improvements; and (4) age-related regression. The point in time or case number in which the outcome of interest begins to stabilize, or plateau, is the inflection point, which delineates the transition from the learning to the proficiency phase [20]. With the continued introduction of novel CN-THA platforms, authors have explored the learning curve associated with their use, and evaluated how patient outcomes are influenced as surgeons gain familiarity with these technologies [21–25]. While insightful, these studies have been inconsistent in the methodologies used to assess the learning curve, making the interpretation of their collective findings unclear. As surgeons will continue to face decisions regarding the implementation of surgical technologies into practice, clarity regarding the early challenges that may be incurred with the use of novel computer navigation platform will be valuable.

Therefore, to comprehensively evaluate the learning curve for adopting CN-THA, a systematic review of current literature was conducted. We aimed to answer: (1) What case load must a surgeon achieve to become proficient in respect to operative time, component placement accuracy, and radiographic outcomes for CN-THA? and (2) How does a surgeon’s initial performance with CN-THA compare to other techniques, such as M-THA?

## Methods

### Search strategy

On June 16, 2023, a search was conducted using PubMed, MEDLINE, EBSCOhost, and Google Scholar to find studies that assessed the learning curve for RA- and CN-THA that were published between January 1, 2000, and June 16, 2023. The Boolean operators “AND” or “OR” were combined with the following keywords and Medical Subject Headings (Mesh): (“Arthroplasty, Replacement, Hip”[Mesh] OR “Arthroplasty, Replacement”[Mesh] OR “total hip arthroplasty” OR “THA”) AND (“Robotics”[Mesh] OR “robotic*” OR “Surgery, Computer-Assisted”[Mesh] OR “Robotic Surgical Procedures”[Mesh] OR “robotic arm” OR “computer navigated”) AND (“Learning Curve”[Mesh] OR “learning” OR “curve” OR “train*” OR “skill*” OR “development” OR “education” OR “proficiency”).

### Eligibility criteria

Eligible articles included studies that had (1) full-text manuscripts in English and (2) evaluated the learning curve in adopting CN-THA. The following articles were excluded from the analysis: (1) case reports, (2) reviews, (3) duplicate articles, (4) gray literature (preprint server articles, posters, and abstracts), and (5) articles not written in English.

### Study selection

**Fig. 1:**
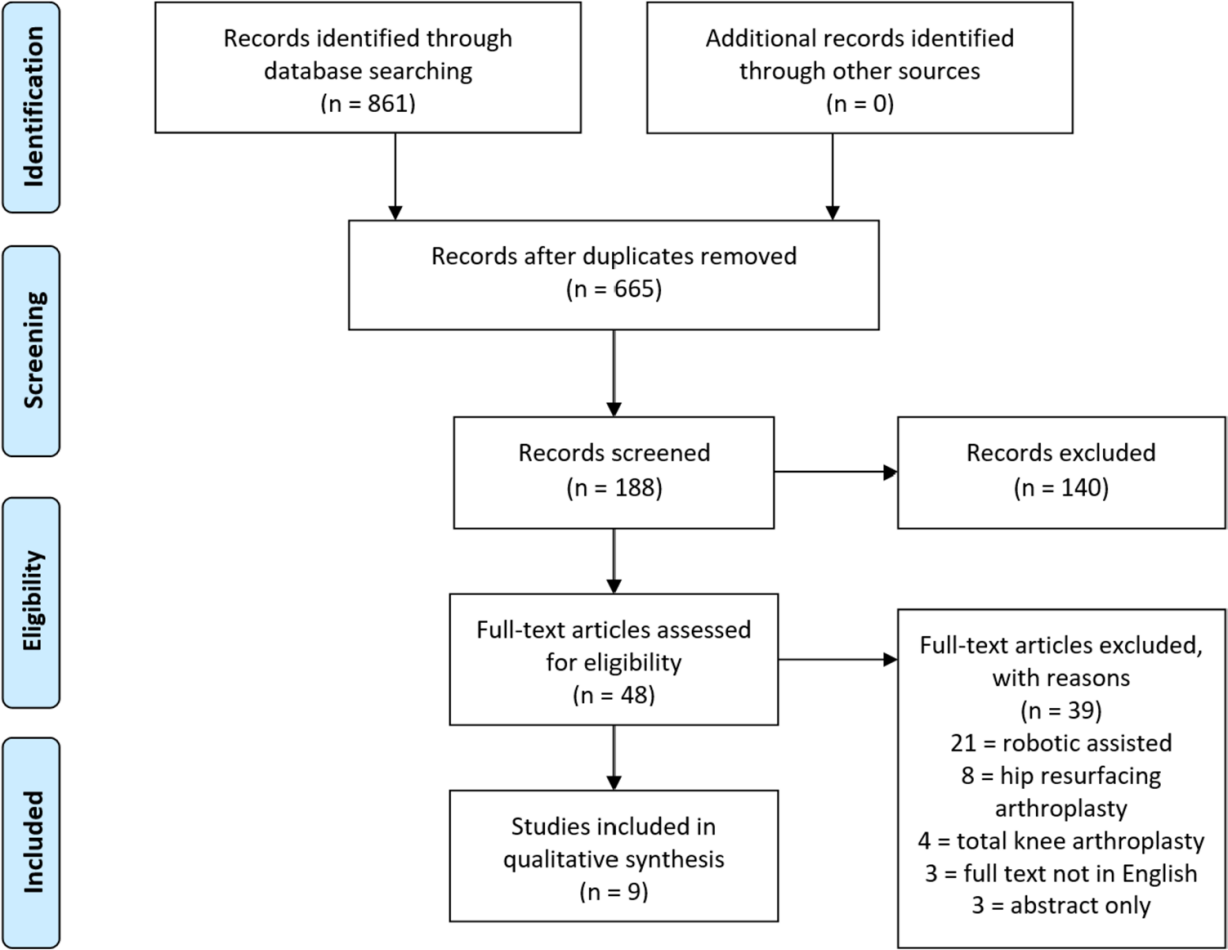
This PRISMA diagram depicts the selection process for article information

The Preferred Reporting Items for Systematic Reviews and Meta-Analyses (PRISMA) guidelines were followed in conducting this review (PROSPERO registration: CRD42023394031, June 27, 2023). After deleting duplicates, 655 articles were returned by the query. Each unique article retrieved via the search term was evaluated for eligibility by two independent reviewers (PAS, JRP). To reach consensus, any differences were consulted with a third reviewer (CJH). Forty-eight papers were eligible for a full-text evaluation after title and abstract screening, with nine meeting all criteria for inclusion in the present analysis. No further studies were found when the reference lists for each article were reviewed (Fig. 1).

### Risk of bias in individual studies

Using the MINORS tool, two independent reviewers (PAS, JRP) evaluated the bias risk [26]. Using 12 criteria regarding the rigor of the study design, outcomes assessed, and follow-up, this verified grading method assigns comparative studies a score between 0 and 24, with higher scores denoting higher quality research. Grading disagreements were settled by reaching a consensus with a third reviewer (CJH). The average MINORS score was 21.3 ± 1.2.

### Outcome measures

Two methods were used to evaluate the learning curve: (1) temporally evaluating a surgeon’s performance over their initial versus later CN-THA cases and (2) comparatively comparing initial CN-THA case outcomes versus outcomes achieved via other THA approaches (namely M-THA). Outcomes of interest included operative time, accuracy of acetabular component placement, radiographic accuracy (LLD and offset), functional outcomes, radiation exposure, and postoperative adverse events. No studies included in the review compared complication profiles during the learning curve. The accuracy of acetabular component placement was assessed with anteversion and inclination (i.e., average values, target value deviations, and safe zone outliers). As the included studies had substantial methodologic heterogeneity, a meta-analysis was not conducted; rather, we conducted a narrative synthesis by presenting and synthesizing key findings. When available, we reported quantitative data for positive findings and qualitative data for negative findings. Likewise, due to substantial heterogeneity in the comparison cohorts among studies, we were unable to visually represent the acetabular component placement accuracy metrics via Bland-Altman plots for both the temporal and comparative assessments of the CN-THA learning curve.

### Study characteristics

Included in the final analysis were a total of nine studies assessing 847 THAs [21–25, 27–30] (Table 1). Six studies assessed the learning curve temporally [21, 23, 25, 27–29] and six assessed the curve comparatively [22–24, 28–30]. Patient characteristics, follow-up timeframes, THA approach, type of acetabular implant, and computer-navigated platform utilized varied among articles (Table 1).

**Table 1:** Characteristics of studies included in the final analysis

Study (year)	Study design	Data source	Sample size	Sex (%M)	Age	BMI (kg/m^2^)	Follow- up	Surgical approach	Acetabular implant	Platform	MINORS
Christ et al. (2018) [24]	Prospective cohort	Single	57	N/R	N/R	N/R	N/R	Posterior	N/R	Intellijoint HIP	20
Gofton et al. (2007) [22]	Prospective cohort	Multi	45^a^	62	N/R	N/R	4–6 weeks	N/R	N/R	Vector vision	24
Inori et al. (2012) [21]	Retrospective cohort	Single	80	16	60	24	N/R	Anterior lateral	Plasmacup	Ortho pilot	21
Kamenaga et al. (2019) [27]	Prospective cohort	Single	75	15	70	24	N/R	Anterior	G7	Hipalign	22
Kolodychuk et al. (2022) [23]	Prospective cohort	Single	159	49	64	27	N/R	Anterior	N/R	Hipalign	21
Najarian et al. (2009) [29]	Retrospective cohort	Single	149	N/R	65	28	N/R	Posterior	N/R	Stryker image guided navigation	20
Suhardi et al. (2021) [30]	Retrospective cohort	Single	90	46	N/R	29	N/R	Posterior	N/R	Intellijoint HIP	20
Thorey et al. (2009) [25]	Prospective cohort	Single	60	42	N/R	29	N/R	Lateral	Threaded SC cup	Ortho pilot	21
Wixson et al. (2005) [28]	Retrospective cohort	Single	132	48	63	29	1 month	Posterior	Varied	Sigma scan pro	21

^a^Simulation-based study—sample size represents training participants

*N/R* not reported, *M *male, *BMI *Body Mass Index, *kg *kilogram, *m* meters, *Multi* multicenter, *MINORS* Methodological Index for Non-Randomized Studies

## Results

### Temporal assessment of the CN-THA learning curve

Of the six studies that assessed the learning curve of CN-THA temporally, five divided the surgeon’s initial cases into cohorts and compared early cases to later [21, 25, 27–29] (Table 2). Two of these studies compared the first 20 procedures to a group of later ones [21, 28], with one showing marked improvements in cup medialization accuracy after 20 cases, but no difference in deviation from planned cup height, anteversion, or inclination, or mean LLD [21]. However, the other study showed marked improvement in anteversion and inclination accuracy, as more components were placed within the target zone after 20 cases (44% vs 87%) [28]. Another study compared the surgeon’s initial 49 cases to 47 cases thereafter, showing a decrease in deviation from planned anteversion (1.04° vs 0.85°) and inclination (0.88° to 0.69°), though no change in operative time [29]. Thorey et al. [25] demonstrated marked differences between intraoperative and radiographic anteversion (15.1° vs 20.9°) and inclination (43.7° vs 47.3°) in cases 1–30, but found no difference in intraoperative versus measured values in cases 31–60. Additionally, navigation time was lessened after 30 cases (13.2 vs 4.8 min). Meanwhile, a remaining study reported a marked decrease in operative time after five cases, though no change in deviation from planned cup inclination or anteversion with experience [27].

**Table 2:** Temporal analysis of the CN-THA learning curve

Study (year)	LC analysis	Key findings
Inori et al. (2012) [21]	Initial 40 cases compared: A. Cases 1–20 B. Cases 21–40	After 20 cases, cup medialization showed marked improvement with a decreased mean discrepancy from 3.1° to 1.6 mm. No difference in deviation from planned cup height, anteversion, inclination, or LLD
Kamenaga et al. (2019) [27]	Initial 75 cases compared: A. Cases 1–25 B. Cases 26–50 C. Cases 51–75	Operative time and navigation time showed a marked decrease after 5 cases and then remained stable for the remaining 70 cases. No difference in deviation from planned cup inclination or anteversion between groups
Najarian et al. (2009) [29]	Initial 96 cases compared: A. Cases 1–49 B. Cases 50–96	Deviation from planned anteversion decreased after 49 cases (1.04° vs 0.85°). Deviation from planned inclination also decreased from 0.88° to 0.69°. Mean blood loss decreased from 520 to 356 mL No difference in operative time
Thorey et al. (2009) [25]	Initial 60 cases compared: A. Cases 1–30 B. Cases 31–60	Navigation time was significantly lessened in the latter 30 cases (13 vs 5 min). The first 30 cases showed a significant difference between intraoperatively planned and postoperative radiographic inclination (43.7° vs 47.3°) and anteversion (15.2° vs 20.9°). However, in the latter 30 cases, there was no difference in intraoperative and postoperatively measured inclination or anteversion, demonstrating improved placement accuracy after 30 cases
Wixson et al. (2005) [28]	Initial 82 cases compared: A. Cases 1–20 B. Cases 21–82	After 20 cases, cup anteversion and inclination accuracy showed marked improvement with experience, as 44% of cups were placed in the combined target zone in the first 20 cases, compared to 87% in the remaining cases
Kolodychuk et al. (2022) [23]	Learning curve was considered completed when the 5-case mean operative time was maintained within the 95% confidence interval of the mean operative time for conventional direct anterior THA	There was a learning curve of 31–35 cases based on operative time

*CN-THA *computer-navigated total hip arthroplasty, *mm *millimeters, *LLD *leg length discrepancy, *mL *milliliters, *THA *total hip arthroplasty

### Comparative assessment of the CN-THA learning curve

Of the six studies that assessed the learning curve of CN-THA comparatively, three compared a surgeon's initial navigated procedures to past conventional procedures they had performed, with one showing marked increases in cups placed within the surgeon’s combined target zone with navigation (30% vs 6%) [28] and another showing fewer ≥ 10° outliers in anteversion (14% vs 21%) and inclination (4% vs 13%) [29] (Table 3). Additionally, one of these studies showed increased operative time for CN-THA (+ 20 min), with little improvement when comparing the early navigated cases (cases 1–49: 128 min), to the later cases (cases 50–96: 124 min) [29]; though another study showed only a modest increase in operative time for CN-THA compared to M-THA (+ 3 min)[24]. Another study compared a surgeon’s initial CN-THA procedures to fluoroscopically guided procedures they had performed, with handheld navigation demonstrating lower deviation from planned inclination (2.9° vs 3.4°) and a longer operative time (92 vs 72 min) over the first 30 cases [23]. After 35 cases, handheld navigation demonstrated lower deviation from planned anteversion (2.0° vs 5.8°) and inclination (1.3° vs 5.4°), lower LLD (1.0 vs 3.4 mm) and offset (1.4 vs 6.1 mm), fewer ≥ 10° outliers for version (0% vs 20%) and inclination (0% vs 15%), and reduced radiation time and dose (dose: 0.6 vs 2.1 mGy; time: 5.3 vs 19.1 s) compared to fluoroscopically guided THA.

**Table 3:** Comparative analysis of the CN-THA learning curve

Study (year)	Comparison	Key findings
Christ et al. (2018) [24]	Single surgeon: initial CN-THA (*n* = 26) vs M-THA (*n* = 31)	The set-up and hands-on utilization of a novel surgical navigation tool required an additional 2.9 min per case (SD: 1.6) compared to M-THA
Wixson et al. (2005) [28]	Single surgeon: initial CN-THA (*n* = 82) vs M-THA (*n* = 50)	Cup inclination of 40° to 45° was achieved in more navigated cases (55 vs 32%). Cup anteversion of 17 to 23° was also achieved in more navigated cases (54 vs 34%). More navigated cases fell into both of these ranges combined (30 vs 6%)
Najarian et al. (2009) [29]	Single surgeon: A. M-THA (*n* = 53) B. initial CN-THA (*n* = 49; cases 1–49) C. later CN-THA (*n* = 47; cases 50–96)	Comparing groups A to B: Navigation had fewer ≥ 10° outliers in anteversion (14 vs 21%) and inclination (4 vs 13%). Operative time was higher in the navigation cohort (128 vs 105 min). No difference in deviation from planned anteversion or estimated blood loss Comparing groups A to C: Navigation had fewer ≥ 10° outliers in anteversion (9 vs 21%) and inclination (4° vs 13%). Operative time was higher in the navigation cohort (124 vs 105 min), although estimated blood loss was lower (356 vs 428 mL)
Kolodychuk et al. (2022) [23]	Single surgeon: initial CN-THA (*n* = 99) vs fluoroscopy-assisted (*n* = 60)	In the learning phase (*n* = 30): Handheld navigation demonstrated lower deviation from planned inclination (2.9° vs 3.4°) and a longer operative time (92 vs 72 min). No difference in deviation from planned anteversion, anteversion outliers, LLD, or radiation time and dose In the proficiency phase (*n* = 64): Handheld navigation demonstrated lower deviation from planned anteversion (2.0° vs 5.8°) and inclination (1.3° vs 5.4°), as well as a lower LLD (1.0 vs 3.4 mm) and offset (1.4 vs 6.1 mm). There were also fewer ≥ 10° outliers for version (0 vs 20%) and inclination (0 vs 15%). Radiation time and dose were lower in the handheld navigation group (dose: 0.6 vs 2.1 mGy; time: 5.3 vs 19.1 s). No difference in operative time
Suhardi et al. (2021) [30]	CN-THA cup placement performed by: A. trials by residents (*n* = NR) B. trials by fellows (*n* = NR) C. Final placement by attending (*n* = 2)	Resident Trials vs Attending: Residents demonstrated greater deviation from planned inclination (5.5° vs 1.3°) and placed more cups outside of the inclination safe zone (23.3 vs 0%). Residents also had greater deviation from planned anteversion (9.6° vs 1.4°). There was no difference in safe zone outliers for version Fellow Trials vs Attending: Fellows demonstrated greater deviation from planned inclination (4.3° vs 1.0°) and anteversion (6.7° vs 1.0°). No difference in proportion of outliers for inclination or version Resident Trials vs Fellow Trials: Fellows achieved fewer outliers from the inclination safe zone (3.3 vs 23%). Residents displayed greater deviation from planned version (9.6° vs 6.7°). There was no difference in outliers from the anteversion safe zone
Gofton et al. (2007) [22]	Medical students and non-orthopedic surgical residents performed simulation-based training in three ways: A. M-THA training (*n* = 15) B. CN-THA training (*n* = 15) C. knowledge-of-results training (*n* = 15)	All groups displayed enhanced accuracy and precision for cup placement inclination and version (*p* < 0.001). The group using computer navigation exhibited superior accuracy and precision in the initial stages of training (*p* < 0.05), maintaining better precision throughout the training process (*p* < 0.05). There was no noteworthy decline in performance when comparing immediate and delayed testing for any of the groups

*CN-THA* computer-navigated total hip arthroplasty, *M-THA* manual total hip arthroplasty, *min* minutes, *LLD *leg length discrepancy, *mm* millimeters, *mGy *milligray, *sec* seconds

## Discussion

Given the variety of CN-THA platforms available and the differing approaches used to evaluate learning curves in the literature, this review aimed to identify patterns in characterizing and evaluating the learning curve. Our analysis of CN-THA demonstrated increased operative times compared to M-THA (3–20 min), though several studies showed improvements could me made over the initial caseload. Additionally, several studies demonstrated a learning curve for component placement accuracy and radiographic outcomes for CN-THA; however, mixed methodologies to analyzing the curve made the exact case number to achieve proficiency unclear. These findings underscore the value of a rigorous, standardized approach to the analysis of surgical learning curves, such as CUSUM analysis, and mitigate concerns for compromised patient outcomes in adopting CN-THA.

### Temporal assessment of the CN-THA learning curve

Several studies investigating the learning curve for CN-THA similarly chose to track the progress of the surgeon over their initial series of navigated procedures. However, CUSUM analysis was not utilized, which may reflect the CN-THA studies having been conducted before CUSUM was popularized. Most studies instead employed a predetermined case number in the series to compare early cases to later ones. The case number chosen to delineate early from later cases was left to the authors’ discretion and varied greatly, between 20 and 50 cases. There were mixed findings regarding the case number required to achieve peak component placement accuracy, which may be a reflection of the limited accuracy of the approach used to analyze the curve as well the fact that different navigation platforms were used. While these studies aimed at estimating an inflection, CUSUM analysis was able to provide an exact case number based on the metric analyzed. Thus, a more standardized approach to the analysis of surgical learning curves in future investigations may allow for more accurate information on the learning process involved with THA technologies and enable direct comparison of available platforms.

### Comparative assessment of the CN-THA learning curve

The use of CN-THA also demonstrated immediate advantages in acetabular component placement accuracy but came with an increased operative time as compared to M-THA. Kolodychuk et al. [23] compared CN-THA to fluoroscopically guided THA and demonstrated that while handheld navigation offered immediate advantages in component inclination accuracy, operative time was initially longer. However, after 35 cases, there was no difference in operative time between approaches and CN-THA began to demonstrate additional advantages, including markedly lower LLD, offset, and radiation time and dose, as well as further improvements in component placement accuracy. Therefore, while CN-THA provides immediate advantages in component placement accuracy and radiographic outcomes, these advantages become more pronounced as experience is acquired. While CUSUM has typically been used to analyze operative time as the outcome of interest, future analyses using alternative outcomes, such as placement accuracy, LLD, offset, and functional outcomes, can provide a clearer understanding of the learning curve required to achieve peak proficiency in CN-THA.

### Limitations

This study had its limitations, many of which resulted from heterogeneity between studies, including the methodologies used to assess the learning curve, navigation platforms, implants, and surgical approaches used, and the statistical analysis of outcomes. As a quantitative synthesis of the evidence was infeasible, and the authors conducted a narrative analysis instead. Additionally, this heterogeneity also prevented direct comparisons between the CN-THA platforms assessed. Furthermore, as the analysis was compromised of observational cohort studies, there is a greater risk of bias in the included studies. Similarly, the intrinsic mean error of each CN-THA platform differs and impacts the overall final placement accuracy of the system, therefore influencing results. Also, none of the included studies assessed complications during the learning curve of CN-THA compared to M-THA, which is a key factor to consider when deciding whether to adopt CN-THA.

## Conclusion

Compared to M-THA, CN-THA offers immediate advantages for implant placement accuracy, and LLD and offset radiographic outcomes. To attain the full extent of these advantages, there is a modest learning curve to achieve peak placement accuracy and radiographic outcomes with CN-THA. Surgeons should expect to experience increased operative times, though marked improvements can be made over a modest caseload. A standardized approach to reporting learning curves, such as CUSUM analysis, can allow for more robust assessment of learning curves associated with various platforms and outcomes of interest. Additional investigation into the complication profile associated with the learning curve of CN-THA is merited to evaluate both the benefits and potential drawbacks of utilizing these intraoperative technologies more fully.

## Data Availability

No datasets were generated or analysed during the current study.
